# 

**Published:** 1844-09

**Authors:** 


					M. V.
?
?
SEPTEMBER, 1844
JNo. L
^ 2. ^ ^,
WBKto&S&l
PUBLISHED
QUARTERLY
PUBLISHED UNDER THE AUSPICI
Jean .
UxmmKi
CHAPIN A, HARRIS M. D,D. D; S.J
EDWARD jMAYftARD, M. D., D.D.S.
JS*-: ^iw-* sSbJlj ?. <t.. ? c ? ?-
JtMOS WESTGOTT, M. D. D.D.S.
-
BAL
) $ 5 ?0 |?er annum, payable in advance. [
This number contains lOi sheets-

				

